# High Carbohydrate, Fat, and Protein Diets Have a Critical Role in Folliculogenesis and Oocyte Development in Rats

**DOI:** 10.1007/s43032-024-01629-1

**Published:** 2024-06-27

**Authors:** Semir Gül, Mehmet Gül, Barış Otlu, Mehmet Erman Erdemli, Harika Gözde Gözükara Bağ

**Affiliations:** 1 Faculty of Medicine, Department of Histology and Embryology, Tokat Gaziosmanpaşa University, Tokat, Turkey; 2 Faculty of Medicine, Department of Histology and Embryology, İnönü University, Malatya, Turkey; 3 Faculty of Medicine, Department of Medical Microbiology, İnönü University, Malatya, Turkey; 4 Faculty of Medicine, Department of Medical Biochemistry, İnönü University, Malatya, Turkey; 5 Faculty of Medicine, Department of Biostatistics and Medical Informatics, İnönü University, Malatya, Turkey

**Keywords:** Carbohydrate, Fat, Folliculogenesis, Infertility, Nutrition, Protein

## Abstract

To date, there is no comparative data on the effects of carbohydrates, fat, and proteins, which are macronutrients, on female reproductive functions. Therefore, in this study, we investigated the effects of diets enriched with carbohydrates, fats, and proteins on folliculogenesis and oocyte development in female rats. 21-day-old rats that were just weaned were divided into 4 groups: control, carbohydrate, fat, and protein. The control group was fed with standard chow and the carbohydrate, fat, and protein groups were fed diets enriched with 75% carbohydrate, 60% fat, and 50% protein for 11 weeks, respectively. It was found that high-fat and high-protein diets caused an increase in the estrous cycle length compared to carbohydrate group (*p* < 0.05). Graafian follicle number decreased in the protein group compared to the control (*p* < 0.05). However, the atretic follicle number was higher in the fat group compared to the control group (*p* < 0.05). In the carbohydrate group, Zp1 was found to be lower than the control and protein groups, Zp2 was found to be lower than the control, and Zp3 was found to be lower than the fat group (*p* < 0.05). While BMP15 was similar between groups (*p* > 0.05), GDF9 was lower in all diet groups compared to the control (*p* < 0.05). Foxo3a was lower in the protein group compared to carbohydrate and control (*p* < 0.05). GAS2 was found to be higher in the control group than the fat group, and higher in the carbohydrate group than the fat and protein groups (*p* < 0.05). FSH, LH, Progesterone, and E2 levels were higher in all three diet groups than in the control (*p* < 0.05). Also, significant differences were observed between the groups regarding adiponectin, resistin, and leptin levels. Taken together, high carbohydrate, fat, and protein intake are associated with impairment of the menstrual cycle, depletion of the developing follicle types, and altered expression of folliculogenesis-specific genes and hormones. Therefore, long-term macronutrient diets may result in shortened reproductive periods and reduced fertilization potential in females in the long run.

**Supplementary Information:**

The online version contains supplementary material available at 10.1007/s43032-024-01629-1.

## Introduction

In recent years, the effects of lifestyle on female reproductive health have received considerable scientific attention [1]. Reproduction and metabolism in humans and animals are closely interrelated and regulated mutually and under the influence of each other [2]. Therefore, it is inevitable that eating habits that directly affect body metabolism have positive or negative effects on the reproductive system. Revealing these effects is also very important in terms of preventing possible infertility problems and increasing fertility.

Puberty is an important developmental event in every individual’s life course. In 1963, Kennedy and Mitra published a study on rats showing that body weight and food intake are important factors of the timing of puberty [3]. Studies have consistently demonstrated that puberty begins at an earlier age over time [4–7]. Early puberty has been associated with increased risks of certain health problems in adulthood, such as obesity, type 2 diabetes, cardiovascular diseases, and various cancers, including breast, endometrial, and prostate cancer [7–11]. A number of molecular pathways and epigenetic mechanisms have been identified as primary components in the modulation of pubertal onset by hormones and nutrition [11]. Macronutrients and hormones that regulate growth and/or influence adipose tissue are metabolic cues that convey nutritional status and stored energy available for sexual maturation, differentiation, and growth [11].

There is no comparative data on the effects of macronutrients carbohydrates, fats and proteins on female reproductive functions. The existence and mechanism of correlation between carbohydrates and reproduction in women of reproductive age has not been fully elucidated and there are many conflicting data in the literature. Some studies have shown that the amount and quality of dietary carbohydrates may be associated with ovulation infertility in nulliparous women [12]. High-fat consumption is a common nutritional problem, with the growing popularity of diets that promote high-fat intake, including the Atkins diet (provides 50–80% of calories from fat), the Paleo diet (provides 28–47% of calories from fat) [13], and the Western diet (provides 40% of calories from fat) [14]. Exposure to a high-fat diet without diet-induced obesity has been shown to lead to reproductive impairment in female mice [15]. High-protein diets, especially those that maintain the absolute number of grams ingested while reducing energy, are a popular strategy for weight loss because of the hunger-controlling effects of protein-based satiety [16]. There are many variants of protein diets such as the Zone diet [17], the CSIRO diet [18] and the Dukan diet [19]. An extensive study has shown an association between animal protein intake and an increased risk of ovulation infertility in a group of healthy women [20].

Follicle and oocyte development is under the influence of environmental, hormonal, and genetic factors. Genes that play a role in normal oocyte development are quite large and some of the genes that play an important role are zona pellucida 1 (Zp1), zona pellucida 2 (Zp2), zona pellucida 3 (Zp3), bone morphogenetic protein 15 (BMP15), growth differentiation factor 9 (GDF9), Foxo3a, and growth arrest-specific protein 2 (GAS2) genes. Zp1, Zp2 and Zp3 proteins participate in the structure of the zona pellucida surrounding the oocyte [21]. BMP15 is encoded in the oocyte and [22] has the functions of supporting the maturation of ovarian follicles in the first stage, preventing advanced stage follicle maturation, determining the number of oocytes that will ovulate, and preventing and proliferation of granulosa cells [23]. GDF9 is encoded from oocytes [24] and is very important to provide the appropriate microenvironment for the oocyte [25]. In the mammalian ovary, Foxo3a protein regulates atresia and follicle growth by inducing apoptosis of granulosa cells [26]. However, Foxo3a protein protects follicle reserve by inhibiting over-activation of reserve follicles and plays an important role in regulating female reproductive period [27]. GAS2, is associated with the cytoskeleton. It was observed that lack of homolog of GAS2 resulted in infertility in some experimental animal models [28].

It is of great importance to know and compare the effects of different food-based diets containing high carbohydrate, protein and fat on the female reproductive system. There is no study in which all macronutrients were studied together and their effects on the female reproductive system were examined and compared in detail. In this context, in our study, the changes in folliculogenesis and oocyte formation processes in terms of estrous cycle, follicle development and reserve, levels of female reproductive hormones and the expression of genes involved in folliculogenesis in female rats were investigated under carbohydrate, protein and fat enriched diets.

## Materials and methods

### Experimental Design

21-day old female *Wistar Albino* rats were used in the study. Rats were kept in cages in groups of five with free access to food and water in rooms with a temperature of 22 ± 2^o^C and seasonal daylight rhythm. 40 rats were randomly selected, divided into 4 groups of 10: Control Group (Cnt): fed with standart maintenance chow, Carbohydrate Group (Ch): fed with 70% carbohydrate enriched chow, Fat Group (F): fed with 60% fat enriched chow, and Protein Group (P): fed with 50% protein enriched chow. Rats were fed with these special feed diets (Table 1) (Research Diets, Inc., 20 Jules Lane, New Brunswick, NJ, 08901 USA) for 11 weeks. The duration of the diet was determined based on previous similar studies [15, 29]. After the rats reached puberty, vaginal smear samples were taken daily to study the estrous cycle pattern. As of the end of the 11th week, the rats which were at diestrus stage of estrous cycle, the phase before ovulation [30, 31], were started to be anesthetized with ketamine (50 mg/kg)– xylazine (10 mg/kg) intraperitoneally and sacrificed after taking of blood samples and ovaries.

**Table 1:** Diet ingredients, formulas, and calories

Diet	Control		70 kcal% Carbohydrate		60 kcal% Fat		50 kcal% Protein	
	g%	kcal%	g%	kcal%	g%	kcal%	g%	kcal%
Protein	24	24	18	18	26	20	49	50
Carbohvdrate	62	64	68	70	26	20	37	38
Fat	5	12	5	12	35	60	5	12
Total		100		100		100		100
kcal/g	3,9		3,9		5,2		3,9	
lngredient	g	kcal	g	kcal	g	kcal	g	kcal
Casein	243,3	973	182,5	730	200	800	507	2028
L-Cystine	3	12	3	12	3	12	3	12
Corn Starch	445	1780	505,6	2022	0	0	181,3	725
Maltodextrin 10	125	500	125	500	125	500	125	500
Sucrose	68,8	275	68,8	275	68,8	275	68,8	275
Cellulose, BW200	50	0	50	0	50	0	50	0
Soybean Oil	25	225	25	225	25	225	25	225
Lard	28	252	28	252	245	2205	28	252
Mineral Mix S10026	10	0	10	0	10	0	10	0
DiCalcium Phosphate	13	0	13	0	13	0	13	0
Calcium Carbonate	5,5	0	5,5	0	5,5	0	5,5	0
Potassium Citrate,1 H20	16,5	0	16,5	0	16,5	0	16,5	0
Vitamin Mix, V10001	10	40	10	40	10	40	10	40
Choline Bitartrate	2	0	2	0	2	0	2	0
FD&C Yellow Dye #5	0,025	0	0,05	0	0	0	0	0
FD&C Red Dye #40	0,025	0	0	0	0	0	0,05	0
FD&C Blue Dye #1	0	0	0	0	0,05	0	0	0
Total	1.045,2	4057	1045	4057	773,85	4057	1.045,2	4057

Each diet was adjusted according to its energy (kcal) percentage in 100 kcal of total energy

g%: gram per 100 gram of total diet

g: gram

### Assessment of Estrous Cycle

Puberty age was noted upon estrous cycle start. Daily vaginal smears were taken onto glass slides by using ~ 5 ml serum physiologique for 60 days and stained with 0.1% cresyl violet. Estrous cycle was divided into 4 stages according to the criteria specified in the literature as proestrus, estrus, metestrus and diestrus [32, 33]. Cycles of 4–5 days in length were considered normal estrous cycles [34], while cycles longer than 5 days were considered abnormal estrous cycles.

### Follicle Count and Morphometric Examination

One ovary of each rat was taken into bouin solution (*n* = 4) for follicle count and into 10% formaldehyde solution (*n* = 6) for imminohistochemical examinations. After 24 h of fixation, ovaries were processed through xylene, alcohol, and melted paraffin serials, and embedded in paraffin blocks. Serial sections of 8 μm thickness at 40 μm intervals were taken from the entire ovary and stained with hematoxylin and eosin (HE). Follicle were grouped according to Pedersen classification [35]. Corpus luteums were also counted at 300 μm intervals. The follicle numbers obtained were multiplied by 5 [(40 μm/8 µm) = 5] to calculate the Total Follicle Count (TFC) for each follicle type and the total corpus luteum numbers in an ovary [33]. Oocyte and follicle diameters were calculated by taking the average of the lengths of two diameters perpendicular to each other from the shortest and longest axes of the oocyte and follicle. All counts and measurements were made manually under bright field microscopy on morphologically normal follicles which contain a prominent oocyte with a nucleus in the center.

### Immunohistochemistry

Ovary sections of 5 μm thickness were first deparaffinized by xylene and alcohol series and brought to distilled water. Imminohistochemical staining was applied as previously described [36] with the primer antibodies, Zp1 (YL-biont YID 5393), Zp2 (YL-biont YID 5394), Zp3 (YL-biont YID 5395), BMP15 (Bioss bs-6612R), GDF9 (Bioss bs-4720R), Foxo3a (YL-biont YID2201), GAS2 (Bioss bs-13289R) and Caspase 3 (NeoMarkers RB-1197-p). Immunohistochemical scoring was made according to the scale of 0; no staining, 1; weak staining, 2; moderate staining, and 3; severe staining. All light microscopy related analyzes were processed by using Nikon Eclipse Ni-U light microscope, DS-Fi3 camera (Nikon Instruments Inc., Melville, NY) and NIS-Elements Documentation 5.02 Image Analysis System (Nikon Corp., Tokyo, Japan).

### Real Time PCR

Primer sequences (Supplementary Table 1) for real time PCR were obtained using the primer-blast online tool [37]. 25 mg ovarian tissue samples (*n* = 5 for each group) were homogenized in 350 µl RTL buffer by Qiagen Tissue Lyser LT device. RNeasy Plus Mini Kit (Qiagen) was used according to the manufacturer’s protocol for RNA isolation. Purity and amount of isolated RNA samples were measured by spectrophotometer (Maestrogene, nano) at 260 nm and 280 nm. Quantiscript Reverz Transcriptase and Quantiscript RT synthesis kit (Qiagen) were used according to the manufacturer’s procedure for gDNA elimination and cDNA synthesis from the obtained total RNA elution. The qPCR analysis performed was a Syber Green-based study, and Qiagen RT2 SyberGreen Master Mix was used. For each 25 µl PCR reaction, qPCR analysis was performed according to the manufacturer’s instructions by using Qiagen Rotorgene Q instrument. Expression levels of 8 genes including 7 target genes (*Zp1, Zp2, Zp3, BMP15, GDF9, Foxo3A, and GAS2*) and one Beta Actin (ACBT) reference gene [38] were investigated.

### Serum Hormone Assays

Blood samples were taken from the right atrium of the heart, centrifuged, and blood sera was separated. FSH, LH, progesterone, estrogen, adiponectin, resistin and leptin hormone levels were measured with ELISA kits specific to each hormone. The ELISA procedure according to the kit protocol (FSH; SunRed − 201-11-0180, LH; SunRed − 201-11-0180, progesterone; SunRed − 201-11-0742, estrogen; SunRed − 201-11-0175, adiponectin; Abbkine - KTE100343, resistin; Abbkine - KTE100300 and leptin; Abbkine - KTE100709) was applied. Within 15 min after the addition of the stop solution, the plates were read at 450 nm wavelength using the ELISA reader device and the results were given as mIU/mL, ng/mL, or µg/L according to the hormone type.

### Statistical Analysis

The compliance of the data to normal distribution was examined using the Shapiro-Wilk test. The data providing the parametric test assumptions were summarized with mean-standard deviation and one-way analysis of variance and then the Tukey HSD pairwise comparison method was used for comparisons. If the data did not satisfy the parametric test, the Kruskal-Wallis test and the Conover pairwise comparison method were used. The results were defined as median, minimum-maximum values. For comparisons of relative gene expression levels were performed with 2^−ΔΔCT^ method using RT² Profiler PCR Arrays & Assays (GenGlobe, Qiagen, Hilden) analysis program. All data were normalized based on the ACTB housekeeping gene (HKG) levels.

## Results

### Estrous Cycle Analysis

**Fig. 1:**
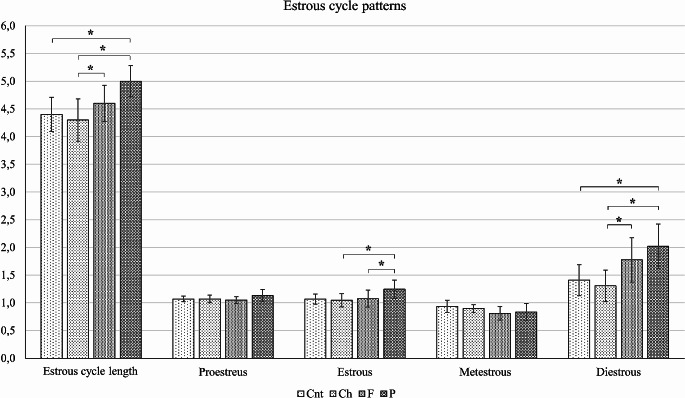
Incidence of estrous cycle phases. Estrous cycle length was found longest in P group which was higher compared to Cnt and Ch groups (*<0.05). F group was also higher compared to Ch group (*<0.05). All groups were similar for proestrus and metestrus pattern. P group was higher compared to Ch and F groups for estrus phase (*<0.05). P group was higher compared to Cnt and Ch, and F group was also found to be higher compared to Ch group for diestrous phase (*<0.05)

There was no statistical significance between the groups for the age of puberty (*p* > 0.05) (data not shown). The estrous cycle pattern for each rat is shown in Supplementary Fig. 1. This three-colored image shows that the red and dark green boxes are more common in the fat and protein groups implying the elongation of the estrous cycle pattern in these groups. In terms of estrous cycle length, carbohydrate group (4.3 ± 0.38 day(d)) was similar to the control group (4.4 ± 0.31 d) (*p* > 0.05), the fat group (4.6 ± 0.33 d) was longer compared to the carbohydrate group (*p* < 0.05), and the protein group (5 ± 0.28 d) was longer compared to both the control and carbohydrate groups (*p* < 0.05) (Fig. 1). Intra-group incidence of each estrous phase (Fig. 1) was also calculated from the Supplementary Fig. 1. Accordingly, prostreus incidence was 1.07 ± 0.05 d in control, 1.07 ± 0.07 d in carbohydrate, 1.05 ± 0.06 d in fat, and 1.13 ± 0.11 d in protein group; estrous incidence was 1.07 ± 0.09 d in control, 1.05 ± 0.12 d in carbohydrate, 1.08 ± 0.15 d in fat, and 1.25 ± 0.16 d in protein group; metestrous incidence was 0.94 ± 0.11 d in control, 0.90 ± 0.07 d in carbohydrate, 0.81 ± 0.12 d in fat, and 0.83 ± 0.15 d in protein group; diestrous incidence was 1.41 ± 0.28 d in control, 1.31 ± 0.28 d in carbohydrate, 1.78 ± 40 d in fat, 2.02 ± 0.40 d in protein group. The incidence of proestrus and metestrus were similar between the groups (*p* > 0.05). The protein group was higher than both carbohydrate and fat groups in terms of estrus phase (*p* < 0.05). For the diestrus phase, fat group was higher compared to the carbohydrate group, and the protein group was higher compared to both control and carbohydrate groups (*p* < 0.05). There was a significant decrease in the protein and fat groups regarding the total number of regular four-days pattern number, 3.2 ± 2.9 and 7.6 ± 4.6, respectively (*p* < 0.05) (Supplementary Fig. 1, light green boxes). Therefore, prolonged and abnormal estrous cycle counts were common in protein and fat groups (Supplementary Fig. 1, dark green and red boxes).

### Follicle Count and Morphometric Examination

**Fig. 2:**
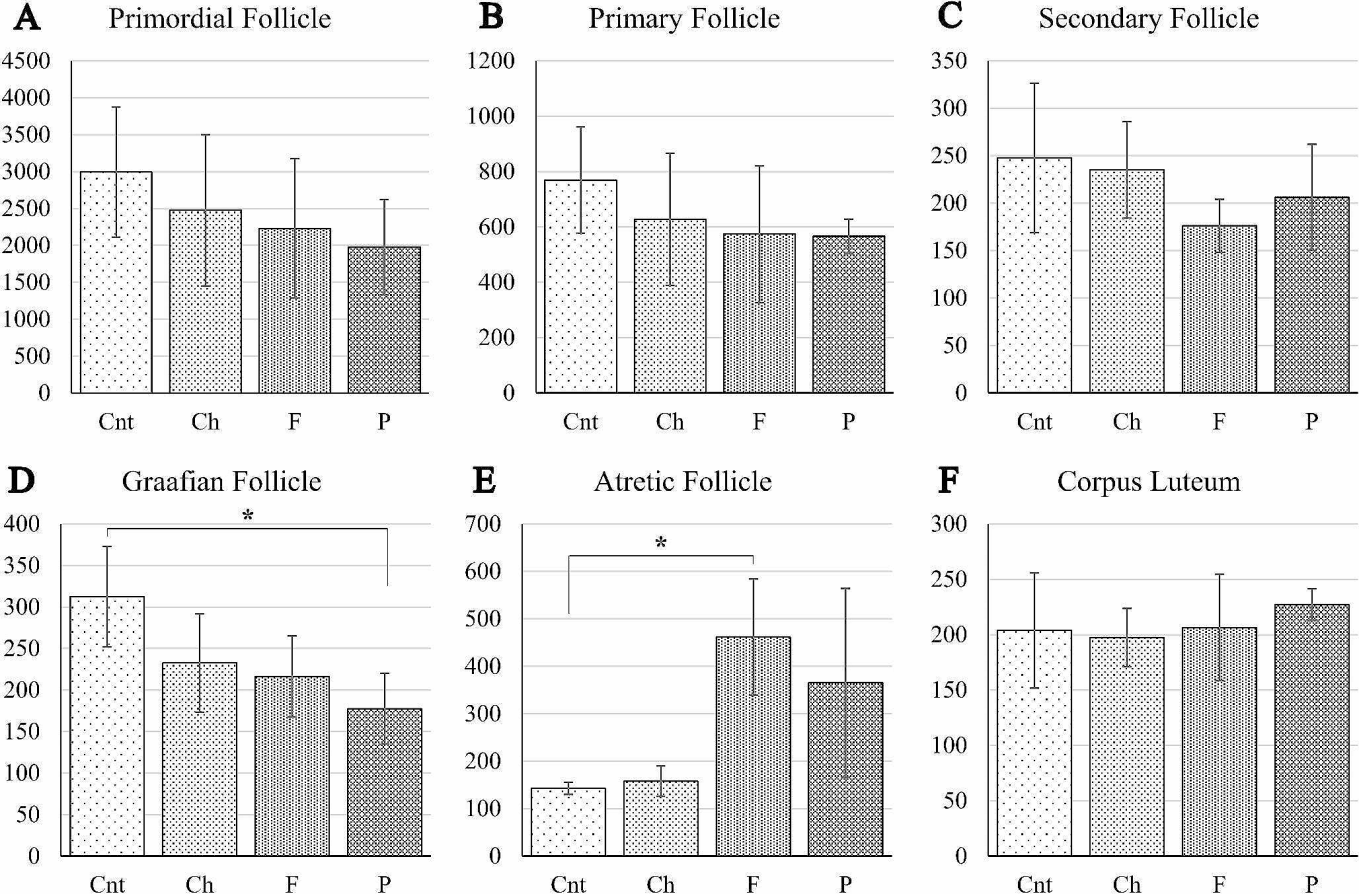
Follicle counts. Although a decreasing trend was observed in all developing follicle types, a significant decrease in terms of graafian follicles was observed only in the P group compared to the Cnt group (*<0.05). F group was higher for atretic follicle number (*<0.05). All groups were similar in terms of corpus luteum number

The average number of follicles and corpus luteum for each group was found as follow; primordial follicles: 2994 ± 881 in control, 2475 ± 1026 in carbohydrate, 2228 ± 944 in fat, and 1976 ± 642 in protein group, primer follicle: 769 ± 192 in control, 628 ± 239 in carbohydrate, 574 ± 248 in fat group, and 566 ± 62 in protein group, seconder follicles: 248 ± 79 in control, 235 ± 51 in carbohydrate, 176 ± 28 in fat, and 206 ± 56 in protein group, graafian follicles: 313 ± 61 in control, 233 ± 59 in carbohydrate, 216 ± 49 in fat, and 178 ± 43 in protein group, atretic follicle: 143 ± 13 in control, 158 ± 32 in carbohydrate, 461 ± 122 in fat, and 365 ± 199 in protein group, corpus luteum: 204 ± 52 in control, 198 ± 26 in carbohydrate, 206 ± 48 in fat, and 227 ± 14 in protein group. Accordingly, for the primordial follicle reserve, there was a gradual decrease in carbohydrate, fat and protein groups, respectively, however this decrease was not statistically significant (*p* > 0.05) (Fig. 2a). Similarly, the decrease in primary and secondary follicle counts, especially in fat and protein groups, wasn’t significant (*p* > 0.05) (Fig. 2b and c). The number of the mature follicle type, the graafian follicle, was found to be significantly decreased in protein group compared to control group (*p* < 0.05) (Fig. 2d). There was an increase in the number of atretic follicle in fat and protein groups, but only the fat group was found to be statistically significant (*p* < 0.05) compared to control group (Fig. 2e). Corpus luteum numbers were similar in all four groups (*p* > 0.05) (Fig. 2f).

The diameter of oocytes and follicles were given in Table 2. The diameter of primary follicle oocyte was found to be higher in protein group compared to all other three groups (*p* < 0.05). For secondary follicle oocyte diameters, fat and protein groups were found lower compared to the carbonhydrate and control groups (*p* < 0.05). The diameter of mature oocytes and the preovulatory follicle oocyte was decreased in carbonhydrate and fat groups compared to control and protein groups (*p* < 0.05). In addition, the diameter of the primary and preovulatory follicles were similar between the groups (*p* > 0.05), however the secondary follicle diameter decreased in protein group (*p* < 0.05).

### RT-PCR Analysis

**Fig. 3:**
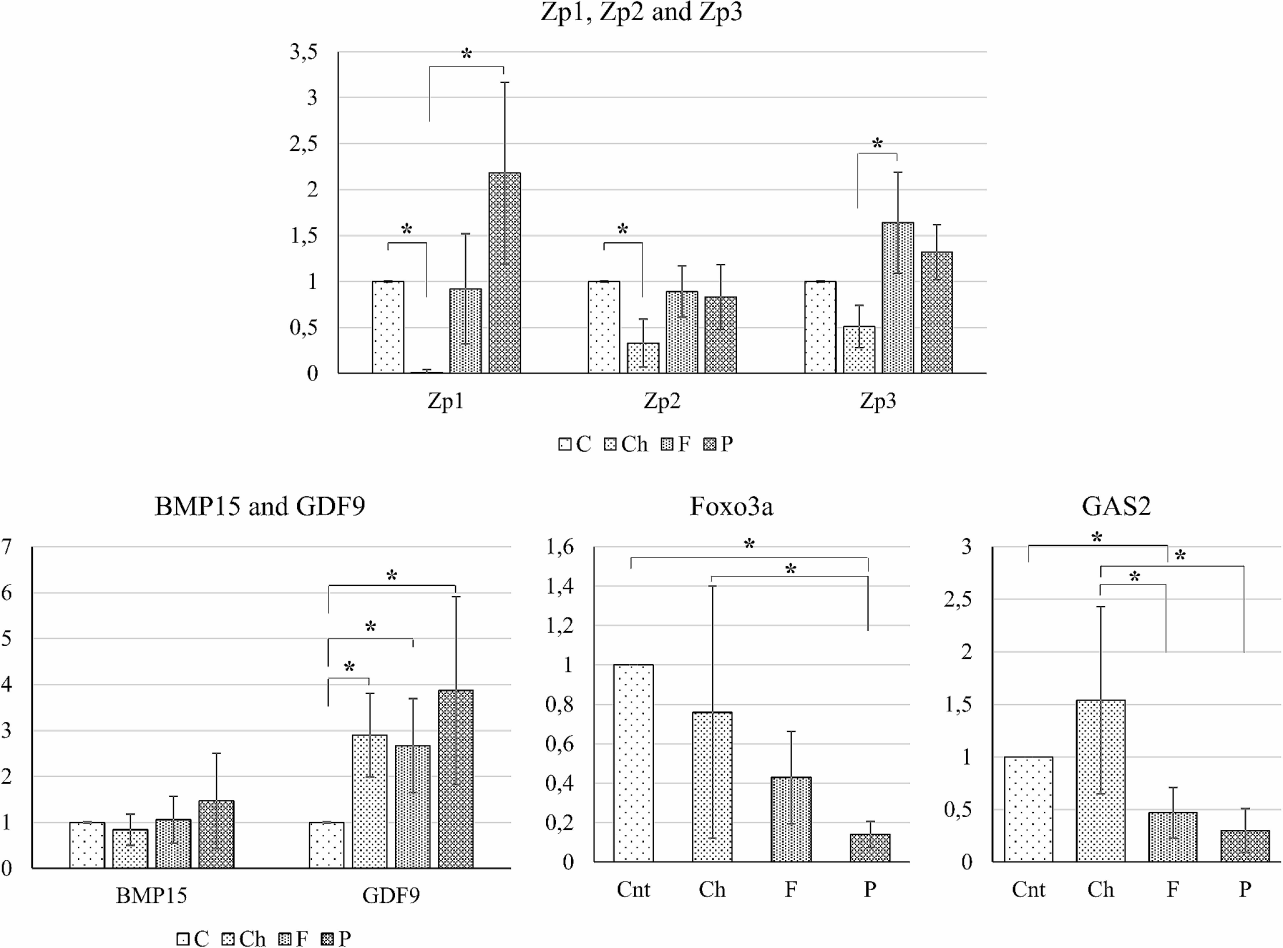
Gene expression profile for folliculogenesis specisic genes. Ch group was decreased (*<0.05) in Zp1 compared to Cnt and P, and in Zp2 compared to Cnt, and in Zp3 compared to F. While BMP15 was similar for all groups, GDF9 was higher (*<0.05) in all diet groups compared to control group. Foxo3a expression was decreased (*<0.05) in P group compared to both Cnt and Ch groups. GAS2 expression was highest in Ch group and it was higher (*<0.05) than the F and P groups. It was also significantly higher (*<0.05) in Cnt group compared to F group

Gene expression levels were given as fold comparing to the control. Compared to the control group, a decrease in the expression of Zp1 (0.01 fold) and Zp2 (0.33 fold) genes (*p* < 0.05) and an increase in the GDF9 gene (2.9 fold) draw attention in carbohydrate group (Fig. 3). Two-fold increase was observed in carbohydrate group for the GAS2 gene, although it wasn’t significant (*p* > 0.05). In the fat group, a significant increase in GDF9 expression (2.67 fold) and a significant decrease in GAS2 expression (0.47 fold) were determined compared to the control group (*p* < 0.05). In addition, in terms of Zp3 expression, the fat group (1.64 fold) was found to be higher than the carbohydrate group (0.51 fold) (*p* < 0.05). For the protein group, an increase in the expression of GDF9 (4.88 fold) and a decrease in the expression of Foxo3a gene (0.23 fold) were detected compared to the control group (*p* < 0.05). A 3.84 fold increase for Zp1 and 2.06 fold increase for Zp3 and 2.04 fold BMP15 expression was also noted in the protein group. Additionally, the protein group was found to be higher in Zp1 (3.84 fold) and lower in Foxo3a (0.23 fold) and GAS2 (0.42 fold) expression compared to Zp1 (0.01 fold), Foxo3a (0.76 fold), and GAS2 (2.05 fold) respectively in the carbohydrate group (*p* < 0.05).

### Immunohistochemical Analysis

**Fig. 4:**
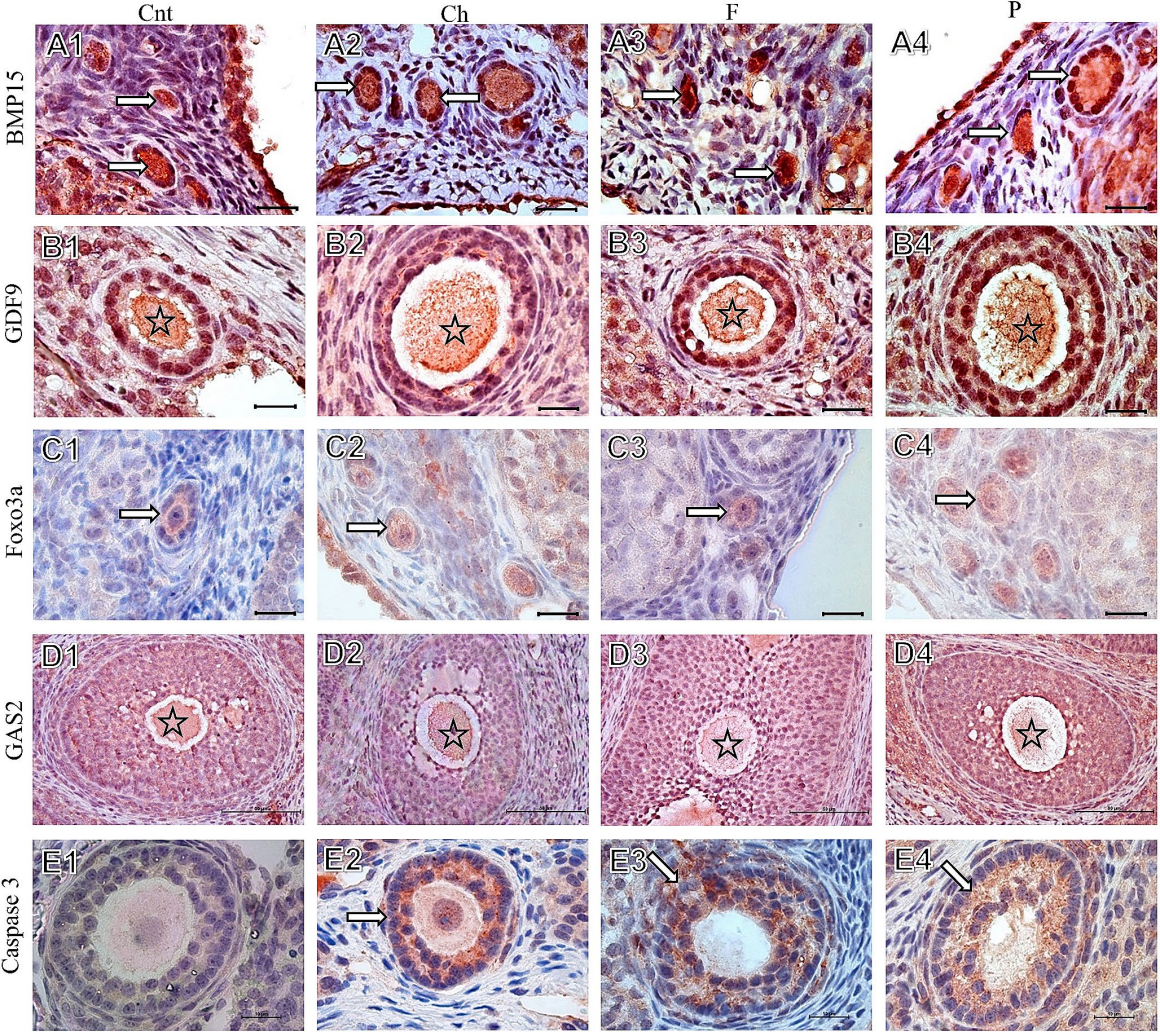
Immunohistochemical staining of BMP15, GDF9, Foxo3a, GAS2, Caspase 3. BMP15 staining in primordial and primer follicles (arrows) were similar in all groups (A1-A4). Oocyte GDF9 staining (stars) score was similar in Cnt and P, and lower than the Ch and F groups (B1-B4). Foxo3a staining in primordial or primer follicles (arrows) were similar in all groups (C1-C4). Oocyte GAS2 staining (stars) intensty were decreased in F and P groups (D3, D4) compared to Cnt and Ch groups (D1, D2). Caspase 3 immunreactivity in follicular cells (arrows) was higher in Ch and F groups (E2, E3) compared to Cnt group (E1). Only representative images were chosen for each protein. Scale bar: 10 μm, IHC, 100x for BMP15, GDF9, Foxo3a and Caspase 3; scale bar: 50 μm, IHC, 40x for GAS2

Immunohistochemical scorings were given in Table 3. There was no significant difference between the groups for Zp1 scoring (*p* > 0.05). In terms of Zp2, carbohydrate group was lower than the other groups (*p* < 0.05). Zp3 scoring was higher in the protein group than the control (*p* < 0.05). When the Zp1, Zp2, Zp3 total scoring was evaluated, carbohydrate group was significantly lower than the control, fat and protein groups (*p* < 0.05). BMP15 scoring was similar in all the groups (*p* > 0.05). GDF9 scoring was similar between the protein and control groups (*p* > 0.05), but it was higher in the carbohydrate and fat groups (*p* < 0.05). When the percentages of Foxo3a+ (positive) primordial follicles were compared, there was no statistical difference between the groups (*p* > 0.05), although there was a decrease tendency especially in the carbohydrate and fat groups. GAS2 protein expression in both oocytes and stromal cells was found to be similar between the control and carbohydrate groups (*p* > 0.05) while the fat and protein groups were lower than both control and carbohydrate groups (*p* < 0.05). There was no significant difference between the groups in terms of caspase 3 reactivity in oocytes (*p* > 0.05). However, caspase 3+ follicle cells were found to be higher in the carbohydrate and fat groups (*p* < 0.05) (Table 3; Fig. 4).

### Hormone Analysis

Hormone levels for each hormon were given in Table 4. Pituitary hormones, FSH and LH, were higher in all diet groups compared to the control group (*p* < 0.001) and there was gradual increase through carbohydrate, fat and protein groups. Ovarian hormones, progesteron and E2, were found to be higher in the carbohydrate, fat and protein groups compared to the control and all three groups were also found to be different from each other (*p* < 0.001). Adipose tissue hormones, adiponectin, resistin and leptin, were measured differently between the groups. Adiponectin levels were found to be lower in the carbohydrate group (*p* < 0.05), higher in the fat group (*p* < 0.05), and similar in the protein group (*p* > 0.05) compared to the control group. Resistin was found to be lower in the fat group (*p* < 0.05), and higher in the protein and carbohydrate groups (*p* < 0.05) compared to the control. There was no significant difference for leptin levels between the fat and control groups (*p* > 0.05), but the carbohydrate and protein groups were higher than the control and fat groups (*p* < 0.05).

## Discussion

Environmental factors such as daylight, climate and food sources are very important for the reproduction of living things and for the offspring to continue to live in a healthy way [39, 40]. Female reproductive system is more sensitive to the negative effects of irregular, unbalanced and malnutrition conditions [40]. However, there is not a comprahensive study related to the nutritional behaviour and fertility in females. In present study, the effects of diets enriched with carbohydrate, protein, and fat were investigated on female reproductive system and infertility.

Malnutrition can affect the age of puberty [3] and cause many metabolic diseases [7–11]. However, no difference was observed in the pubertal age of the rats between the groups in the present study. Imbalanced intake of macronutrients may also cause serious health problems such as infertility due to estrous irregularities [41]. Mandl et al. measured the regular estrous cycle length in rats as 4.4 days [34]. Similarly, in our study, the mean estrous cycle length was found to be 4.4 days in the control group. However, a prolonged estrous cycle was detected in the protein (5.0 days) and fat groups (4.6 days). Similar to our findings, it was shown by Lilia BS et al. that 18.3% of obese women develop oligomenorrhea (prolonged estrous cycle) and 11.7% develop amenorrhea (shortened menarche phase) [42]. According to the our observations, the shortening of estrous indicates that protein and fat-based diet may cause amenorrhea, while the lengthening of diestrus indicates that protein and fat-enriched diet may cause oligomenorrhea. In the study conducted by Zheng T et al., it was shown that diets with high protein and fat content weaken the immune system [43]. Since the diestrus is the regeneration phase of the uterine endometrium, a significant prolongation of this phase, in our study, suggests that it may be related to the weakening of the immune system due to long term protein- and fat-enriched nutrition.

In a study concluded by Teleni et al., it is stated for cattle that protein diet causes an increase in blood glucose levels and increases ovulation due to the high energy associated with it [44]. However, in a study conducted on humans by Irene S et al., high protein diet caused a decrease in the number of antral follicles [45]. In other words, some studies show that a high protein diet induces folliculogenesis, and some studies show that although it induces folliculogenesis, it causes the folliculogenesis process to be incomplete. In addition, many studies with a high-fat diet have shown that a high-fat diet also causes a decrease in follicle reserve [46]. Similar to the literature, in our study, the decrease tendency in primordial follicle reserve in protein and fat groups, may be due to advanced induction of folliculogenesis. However, the decrease in the number of healthy graafian follicles and the increase in the number of atretic follicles and caspase-3 immunoreactivity indicate that the fat and protein induced folliculogenesis process cannot be completed properly. Although high protein and fat diets induce folliculogenesis, the most obvious evidence that it negatively affects the normal folliculogenesis process is the number of atretic follicles. In a study conducted by Wang Na et al., it was shown that a high-fat diet increased the number of follicles induced for folliculogenesis, but also significantly increased the number of atretic follicles compared to the control [47]. Similarly, the number of atretic follicles was found to be higher in the fat group than in the control group in our study. Furthermore, corpus luteum numbers were found to be similar between the groups, which implies that similar number of ovulation occurs in all diet groups although folliculogenesis is induced in fat and protein groups, representing the impaired folliculogenesis process.

In a study conducted by Akbariasbagh F et al. on oocytes obtained from humans, it was shown that the fertilization rate of low-volume oocytes was significantly lower than the fertilization rate of normal-volume oocytes [48]. In another study conducted on goats by Begona A et al., it was reported that healthy embryos did not form as a result of fertilization of oocytes with a diameter of less than 110 μm and excessively large (> 135 μm) oocyte volume also caused a decrease in the number of healthy developing embryos [49]. In short, if the oocyte volume is too small or large, it negatively affects fertility. In our study, for the diet groups, significant differences were observed in oocyte diameters in primary, secondary and graafian follicles compared to the control group. This suggests that long-term carbohydrate, fat and protein-based nutrition may adversely affect fertilization and embryo development due to abnormality in oocyte diameter.

In a study conducted by ZJ Lan et al. on mice, it was shown that overexpression of BMP15 and GDF9 genes caused hypofertility by decreasing the number of offspring and prolonging the estrous cycle [50]. Again, in another study by Y Peng et al. in mice, it was shown that BMP15 and GDF9 proteins play a heterodimeric role together and a very specific level of expression is required for normal oocyte development [47, 51]. These studies show that the decrease in the expression levels of these two proteins, as well as their higher than normal levels, negatively affect follicle and oocyte development. When the data in our study are evaluated, it is seen that there is an inverse correlation between the amount of BMP15 and GDF9 expression, and the number of advanced follicles and oocyte diameter. In protein group, while both BMP15 and GDF9 RNA levels are higher than the other groups, it is seen that all developing follicle types are the lowest in this group in terms of the number of follicles, excluding secondary follicles. This shows that the over-expression of BMP15 and GDF9 proteins may cause a decrease in the number of follicles inversely. In addition, in a study by JZ Lan et al., it is stated that overexpression of these proteins causes an increase in estrous cycle lengths [50]. When the estrous cycle data we obtained are examined, it is seen that the longest cycle duration is in the fat and protein groups, that is, in the groups with high BMP15 and GDF9 RNA levels. In other words, BMP15 and GDF9 proteins are expressed at a high level as a result of fat and protein diet, which negatively affects normal oocyte development by causing both a decrease in the number of follicles and a prolongation of the estrous cycle.

Considering the mechanism of action of Foxo3a, it is seen that it suppresses BMP proteins in primordial follicles and regulates follicle development by this mechanism [52]. According to the data we obtained, considering the Foxo3a mechanism of action, the decrease in Foxo3a expression in fat and protein groups results in an increase in the expression of BMP15 and GDF9, thus inducing reserve primordial follicles for further follicular development. Therefore, it is thought that the main molecular reason underlying the decrease tendency in follicle reserves in the fat and protein groups, may be the increased expression of BMP and GDF9 as a result of decreased Foxo3a protein. For that reason, the decrease in Foxo3a expression level as a result of unbalanced nutrition shows that it may cause both a decrease in the number of reserve follicles and inhibition of healthy follicle development in the ovary, resulting in infertility. The mechanism by which carbohydrate, fat and protein macronutrients suppress Foxo3a and whether they directly affect BMP15 and GDF9 independently of Foxo3a is a situation that needs to be clarified by new studies.

Besides many cells, GAS2 protein is also synthesized in oocyte and follicle cells, being more prominent in ovarian stromal cells, and is a necessary protein for normal follicle development [53]. Studies in Drosophila, pigs and mice have shown that GAS2 mutation results in sterility [28, 53]. The decrease in GAS2 expression we obtained in our study reveals another factor underlying the decreasing tendency in total follicle numbers in fat and protein groups. However, since the effect of GAS2 on folliculogenesis has been newly defined, there is not enough information about its mechanism of action yet [53].

The reduction in oocyte diameter at seconder and graafian oocytes in the carbohydrate group is thought to be related to the decreased expression of Zp1, Zp2 and Zp in this group. In addition, in the electron microscopic examinations (Supplementary Fig. 2), intense structural deformations were detected in the zona pellucida of the oocytes in this group. When we look at the relationship between the increase in the expression level of Zp1 and Zp3 genes in the protein group and the oocyte volume, it is seen that the oocyte diameter is abnormal from the primary follicle to the preovulatory follicle stage in the protein group. In other words, overexpression of Zp genes may cause anomalies in oocyte volume. In addition, intense structural deformations were detected in the zona pellucida structures, which were examined in electron microscopic sections of the protein group, similar to the carbohydrate group (Supplementary Fig. 2). It is thought that changes in the expression profiles of Zp proteins depending on the type of nutrition may also negatively affect fertility, for reasons such as the role of the zona pellucida in determining the oocyte volume, which has an important effect on fertilization by determining the borders of the oocyte, its role in sperm binding during fertilization and its functions in the prevention of polyspermia.

Increase in FSH and LH hormone levels in carbohydrate, fat and protein groups suggest that the induction of more primordial follicles for development in these three groups may be the reason for the decreasing trend in follicle reserve, respectively. In a study conducted by Chisato Nagata et al., it was shown that high fat intake increases the estrogen level [54]. In another study conducted by Akamine EH et al., it was stated that a high-fat diet increased the level of progesterone [55]. Considering the estrous cycle findings obtained in our study and the role of the estrogen hormone in the regulation of the estrous cycle, it is thought that the estrous irregularities and high abnormal estrous cycle numbers observed especially in the fat and protein groups may be the result of advanced synthesis of the estrogen hormone in those groups. As a matter of fact, studies have shown that the increase in progesterone and estrogen causes an increase in the length of the estrous cycle and disruption of the estrous cycle order [55].

Studies have aslo shown that adiponectin values ​​decrease, and resistin and leptin values ​​increase in obese animals [56]. In our study, adiponectin level was higher, resistin level was lower, and leptin level was similar to the control group, since the rats in the fat diet group did not reach the level of overweight or obesity yet. Leptin is known to be induce both sex hormones of pitiutary gland and ovary [57]. Similary, FSH, LH, E2 and progesteron were found to be increased in carbohydrate and protein groups in which leptin levels were also higher. One of the reason for induction of folliculogenesis especially in protein group may be due to high leptin induced over expression of genes such as BMP15, and GDF9, and hormones such as FSH, LH, E2 and progesteron playing role in folliculogenesis process. Studies have shown that changing the levels of adipose tissue hormones, the adiponectin, resistin and leptin, indirectly play a role in processes such as estrous cycle pattern, oocyte development and ovulation. However, there is insufficient information on how adipose tissue hormones affect nutrient consumption, which is important in oocyte development [56] and requires more extensive experimental studies.

Taken together, fat and protein-based nutrition cause estrous cycle irregularities, abnormal increase or decrease in the expression of folliculogenesis-specific genes and proteins, and changes in the levels of pituitary and ovarian hormones that have a direct effect on oocyte development. As a result of these changes, in the long term, the rapid decrease in primordial follicle and oocyte reserve, suppression of follicle development and decrease in oocyte quality may cause shortening of the reproductive period and decrease in fertilization potential. However, in order to fully understand the relationship between nutrition and fertility, more comprehensive and detailed experimental studies are needed to elucidate the mechanism-based relationships between nutrition types and changes in proteins and hormones involved in folliculogenesis.

**Table 2:** Follicle and oocyte diameter measurements (µm)

	Primary Follicle Oocyte	Primer Follicle		Secondary Follicle Oocyte	Secondary Follicle	Preovulatory Oosit	Preovulatory Follicle
Cnt	10.69 ± 0.74^a^	19.54 ± 1.20		28.16 ± 0.61^a^	56.73 ± 2.59^a^	42.28 ± 1.76^a^	300.08 ± 34.08
Ch	10.13 ± 0.96^a^	18.97 ± 1.45		24.71 ± 0.49^a^	52.29 ± 5.96^a^	37.65 ± 1.31^b^	264.88 ± 14.48
F	10.71 ± 0.73^a^	19.99 ± 1.66		24.88 ± 0.69^b^	50.58 ± 1.63^a^	39.25 ± 1.85^b^	275.66 ± 29.04
P	11.28 ± 0.25^b^	20.36 ± 0.48		22.86 ± 2.61^b^	44.24 ± 8.68^b^	40.06 ± 2.13^a^	300.86 ± 36.28
p-value	< 0.05	> 0.05	< 0.05		< 0.05	< 0.05	> 0.05

* Same letters represents similarity (*p* > 0.05) and different letters represents significant difference (*p* < 0.05)

**Table 3:** Immunohistochemical scoring for folliculogenesis spesific proteins and caspase 3

	Zp1	Zp2	Zp3	BMP15	GDF9	Foxo3a	GAS2	Caspase 3
Cnt	2.05 (1.95–2.33)	1.94 (1.53–2.50)^a^	2.19 (1.60–2.43)^a, b^	2,6 (2.10–2.75)	2.4 (2.40–2.50)^a^	75 (35–100)	2.46 (2.20–2.67)^a^	1.33 (0.86–1.45)^a^
Ch	1,77 (1.33–1.86)	1.36 (0.63–1.56)^b^	1.69 (1.50–1.75)^a^	2,65 (2.30–2.70)	2.6 (2.50–2.60)^b^	51 (33–91)	2.43 (2.33–2.62)^a^	5.17 (3.95–6.91)^b, c^
F	2,21 (1.82–2.80)	2.02 (1.90–2.22)^a^	2.21 (2.13–2.50)^b, c^	2,40 (1.70–2.80)	2.7 (2.60–2.80)^c^	56 (17–83)	2.17 (1.71–2.71)^b^	6.04 (5.12–6.48)^b^
P	2,24 (1.83–2.38)	2.26 (1.79–2.44)^a^	2.53 (2.17–2.80)^c^	2,70 (2.70–2.80)	2.35 (2.25.2.50)^a^	74 (67–78)	1.78 (1.50–2.07) ^b^	3.51 (1.13–5.33)^a, c^
p-value	> 0.05	< 0.05	< 0.05	> 0.05	< 0.05	> 0.05	< 0.05	< 0.05

*Same letters represents similarity (*p* > 0.05) and different letters represents significant difference (*p* < 0.05)

**Table 4:** Pituitary, ovarian and adipose tissue hormone levels

	Pituitary Hormones		Ovarian Hormones		Adipose Tissue Hormones		
	FSH mIU/mL	LH mIU/mL	Progesteron (ng/mL)	E2 (ng/L)	Adiponectin (µg/L)	Resistin (µg/L)	Leptin (µg/L)
Cnt	10.53 ± 0.26^a^	29.17 ± 0.67^a^	2.53 ± 0.03^a^	141.45 ± 3.31^a^	4.22 ± 0.81^a^	5.66 ± 0.16^a^	2.06 ± 0.14^a^
Ch	11.67 ± 0.37^b^	31.06 ± 0.63^b^	4.39 ± 0.26^b^	153.12 ± 5.01^b^	2.45 ± 0.33^b^	8.53 ± 0.33^b^	4.16 ± 0.13^b^
F	13.10 ± 0.34^c^	33.41 ± 0.68^c^	7.45 ± 0.36^c^	174.20 ± 2.52^c^	9.68 ± 0.87^c^	5.08 ± 0.36^d^	2.08 ± 0.07^a^
P	14.23 ± 0.62^d^	36.20 ± 0.90^d^	5.15 ± 0.07^d^	159.57 ± 2.08^d^	3.60 ± 0.25^a^	7.38 ± 0.38^c^	3.35 ± 0.37^c^
p-value	< 0.001	< 0.001	< 0.001	< 0.001	< 0.001	< 0.001	< 0.001

*Same letters represents similarity (*p* > 0.001) and different letters represents significant difference (*p* < 0.001)

## Electronic Supplementary Material

Below is the link to the electronic supplementary material.

Supplementary Material 1

Supplementary Material 2

Supplementary Material 3

Supplementary Material 4

## Data Availability

The data that support the findings of this study are available on request from the corresponding author.
