# Supplementary figures and images for: High Carbohydrate, Fat, and Protein Diets Have a Critical Role in Folliculogenesis and Oocyte Development in Rats
Source: Reprod Sci. 2024 Jun 27;31(10):3215–27. doi: 10.1007/s43032-024-01629-1 (PMC11438621; doi:10.1007/s43032-024-01629-1)

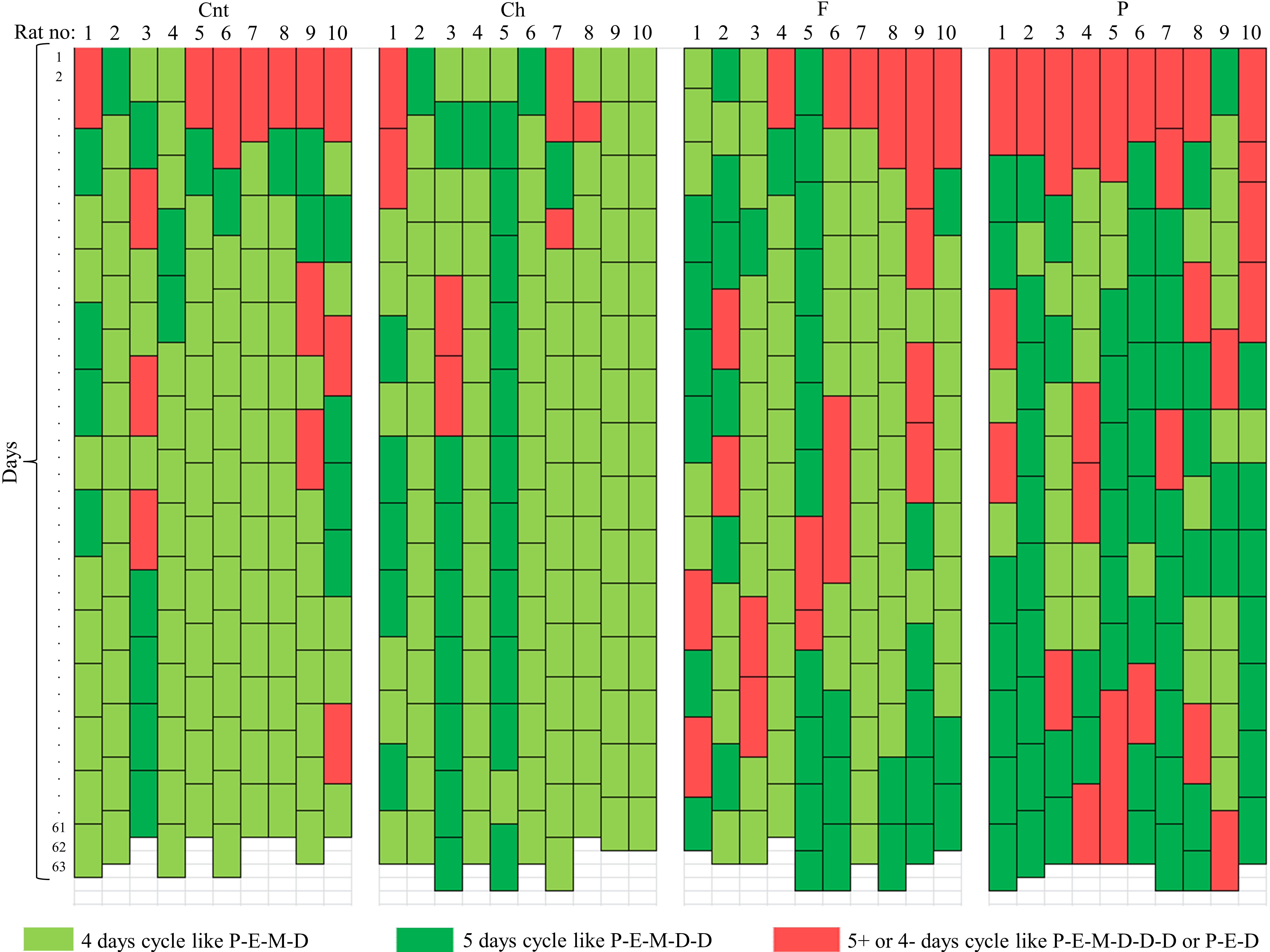

Supplement: Supplementary file 1 — Supplementary Material 1 [file 43032_2024_1629_MOESM1_ESM.jpg]

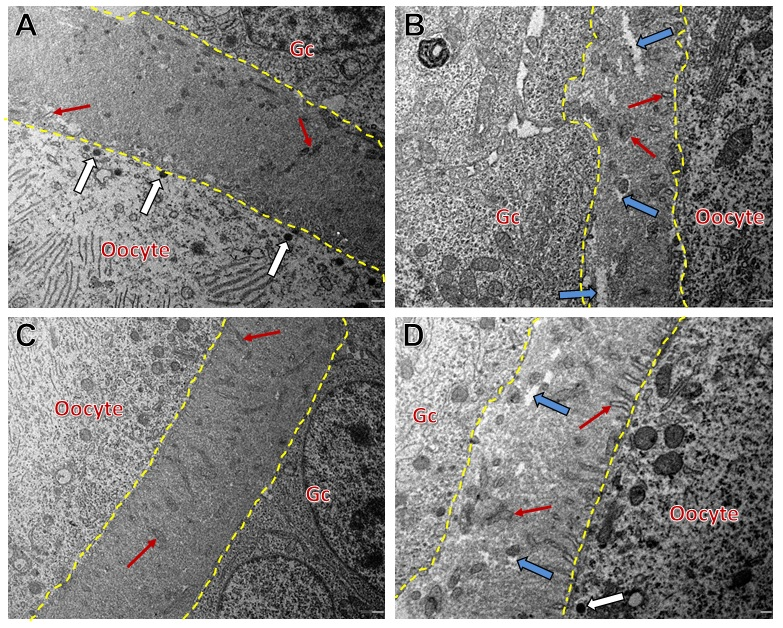

Supplement: Supplementary file 2 — Supplementary Material 2 [file 43032_2024_1629_MOESM2_ESM.png]
