# Supplementary material for: High Carbohydrate, Fat, and Protein Diets Have a Critical Role in Folliculogenesis and Oocyte Development in Rats
Source: Reprod Sci. 2024 Jun 27;31(10):3215–27. doi: 10.1007/s43032-024-01629-1 (PMC11438621; doi:10.1007/s43032-024-01629-1)
Supplement: Supplementary file 3 — Supplementary Material 3 [file 43032_2024_1629_MOESM3_ESM.docx]

Supplementary Table I. Primers for RT-PCR analyses

| Gene code | Sequence (5’ to 3’)  (F: forward, R: reverse) |
| --- | --- |
| ACBT (F) | TCGAGTCGCGTCCACC |
| ACBT (R) | GGGAGCATCGTCGCCC |
| Zp1(F) | GCCACCTTCACTCTCCTTGA |
| Zp1 (R) | CGTCGATGCCTTGCTATCTC |
| Zp2 (F) | GGCTCTCCAGCCTGATCTACT |
| Zp2 (R) | TGGGAGGCTAACCGTCATTG |
| Zp3 (F) | AGACTTCCCAGAGTTGGTTG |
| Zp3 (R) | TGACATCAGCTTCATCCGTCA |
| BMP15 (F) | GATAAAGCCGTCAGCCAGTGC |
| BMP15 (R) | AGGTTCCACATGGCAGGAGAG |
| Gdf9 (F) | GGCTCCCAGCAACCAGATGA |
| Gdf9 (R) | GAGGAGGAAGCAGCGGAGTT |
| Foxo3a (F) | CATCTCAAAGCTGGGTGCCA |
| Foxo3a (R) | GATGGCGTGGGAGTCACAAA |
| GAS2 (F) | AACAAGCCTGCCAAGACCCT |
| GAS2 (R) | AGACACACTTCTCGGGGCTG |
