# Supplementary material for: High Carbohydrate, Fat, and Protein Diets Have a Critical Role in Folliculogenesis and Oocyte Development in Rats
Source: Reprod Sci. 2024 Jun 27;31(10):3215–27. doi: 10.1007/s43032-024-01629-1 (PMC11438621; doi:10.1007/s43032-024-01629-1)
Supplement: Supplementary file 3 [file 43032_2024_1629_MOESM4_ESM.docx]

İNÖNÜ UNIVERSITY

FACULTY OF MEDICINE

EXPERIMENTAL ANIMALS ETHICS COMMITTEE DECISION

Meeting date	: 02.11.2017
Meeting location	: Meeting room, Faculty of Medicine, Mal
Research protocol no	: 2017/A-57
Species of animals to be used in the experiment	: Rat
Strain of animals to be used in the experiment	: *Wistar Albino*
Gender of animals to be used in the experiment	: Female
Number of animals to be used in the experiment	: 60
Age and weight of animals to be used in the experiment	: 21 days

The file of the study with protocol number 2017/A-57, titled "Investigation of the effects of different nutrition types on the development of obesity, folliculogenesis and oocyte quality", conducted by prenciple investigator Prof. Dr. Mehmet Gül, one of the faculty members of the Faculty of Medicine, was examined.

It was unanimously decided that there were no ethical drawbacks in conducting the said study, provided that the research protocol was fully complied with, the matters specified in the İnönü University Faculty of Medicine Experimental Animals Ethics Committee Directive were fulfilled, and the responsibility belonged to the researchers.

Prof. Dr. Davut ÖZBAĞ<br>Başkan	Katılmadı<br>Prof. Dr. Tayfun GÜLDÜR<br>Başkan Yardımcısı	Prof. Dr.Metin ATAMBAY<br>Üye
Prof. Dr. M. Çağatay TAŞKAPAN<br>Üye	Prof. Dr. Başak KAYHAN<br>Üye	Doç. Dr. Şengül YÜKSEL<br>Üye
Vet.Hek.Özge ÖZYALIN<br>Üye	Katılmadı<br>Akın ÖZ<br>Sivil Üye	Katılmadı<br>Dr.Mehmet DENİZ<br>Sivil Üye
